# Physical, sexual and overall reported interpersonal violence against adult out-patients with severe mental illness under remission, receiving healthcare at Butabika hospital: A cross-sectional study

**DOI:** 10.1371/journal.pone.0314402

**Published:** 2024-12-02

**Authors:** Edgar Innocent Guma, Paul Bangirana, Caroline Birungi, Patrick Ocen, Zahra Morawej, Noeline Nakasujja

**Affiliations:** 1 Department of Psychiatry, College of Health Sciences, Makerere University, Kampala, Uganda; 2 Faculty of Medicine, Department of Psychiatry, Hubert Kairuki Memorial University, Dar es Salaam, Tanzania; University of Toronto, CANADA

## Abstract

Interpersonal violence is a significant public health and human rights concern. People living with severe mental illness are especially vulnerable. The Sustainable Development Goals 2030 Agenda aims to end violence. To this end, we gathered information on the prevalence and factors associated with interpersonal violence among one of the most impacted groups: individuals with severe mental illness at Butabika Hospital in Kampala, Uganda. We conducted a cross-sectional study in 2020, including individuals 18 years or older. Data was collected through a socio-demographics questionnaire and nine questions from the modified My Exposure to Community Violence Questionnaire. The assessment evaluated physical or sexual violence experience, frequency, and perpetrator identity. The data was analyzed using STATA version 12 through simple logistic regression to determine the correlation between a single exposure and the outcome of interest, with a significance level of 5%. Among 385 participants, the past year prevalence of overall reported interpersonal violence was about 34%, while physical and sexual reported interpersonal violence were approximately 29% and 11%, respectively. Participants who had perpetrated physical violence had higher odds of experiencing reported interpersonal violence. With increasing age, the odds of experiencing reported interpersonal violence decreased; compared to those aged 18–24 years, those aged 35–44 years had AOR = 0.31 (95% CI: 0.14–0.70, p = 0.005), and those aged 45 years and above had AOR = 0.34 (95% CI: 0.15–0.80, p = 0.013). Reported interpersonal violence was high among the participants. While individuals of older age had lower odds of reporting interpersonal violence, those who had perpetrated physical violence in the past year had higher odds. Screening for interpersonal violence among young patients is recommended. Psycho-education on preventing physical violence should be provided, especially to younger adults. The Ministry of Health should address violence against individuals with mental illness through mass sensitization. A prospective study could investigate risk and protective factors.

## Introduction

Violence is a significant global issue that impacts all nations and communities [1]. Defined by the World Health Organization as "the intentional use of physical force or power, threatened or actual, against oneself, another person, or a group or community, resulting in injury, death, psychological harm, maldevelopment, or deprivation", it encompasses self-directed, interpersonal, and collective forms [1–3]. Self-directed violence includes suicidal behavior and self-harm, while interpersonal violence encompasses violence inflicted by an individual or small group, including family and intimate partner violence as well as community violence. Collective violence is committed by larger groups such as states, political organizations, militias, and terrorist groups [1]. Among these, interpersonal violence stands out due to having the most widespread impact, posing significant public health and human rights challenges [4]. It is a leading cause of mortality among young adults and adolescents and leaves survivors vulnerable to long-term emotional, behavioral, and physical health issues [5]. According to the WHO, there are five main types of violence: physical, sexual, psychological, deprivation, and neglect [1]. This study specifically focuses on physical and sexual violence for a few key reasons: First, physical and sexual violence lead to specific physical injuries and psychological trauma, requiring separate studies to address their distinct health outcomes: For instance, the World Health Organization notes that sexual violence can result in reproductive health issues and sexually transmitted infections, which are typically not associated with psychological violence or neglect [1,3]. Furthermore, the National Institute of Mental Health (NIMH) highlights that trauma from physical and sexual violence can result in conditions such as post-traumatic stress disorder (PTSD), depression, and anxiety, which require specific treatments different from those for neglect or psychological abuse [6]. Recognizing how physical and sexual violence affects people with severe mental illness (SMI) helps tailor effective interventions. Thornicroft advocates for distinct strategies to address the different types of violence among people with severe mental illness to better understand their unique risk factors, health impacts, legal implications, and public perception, ultimately aiming for more effective and targeted interventions [7].

In 2014, over 50,000 deaths worldwide were attributed to violence, with a disproportionate 83% occurring in low- and middle-income countries (LMICs) [8]. Within these LMICs, 91.4% of violence-related deaths are due to interpersonal violence [5]. Like other vulnerable groups, such as the homeless and individuals with developmental disabilities [9,10]. Individuals with severe mental illness (SMI), such as schizophrenia, bipolar disorder, and severe major depression, face heightened likelihood of experiencing physical and sexual interpersonal violence, including rape and sexual assault [11–13]. They are also more likely not only to experience but also to perpetrate violence compared to the general population [14,15]. Severe mental illness encompasses specific psychiatric disorders such as psychotic disorders and major affective disorders, marked by enduring cognitive, behavioral, and emotional symptoms that significantly impair daily functioning [16]. Psychotic symptoms, such as delusions involving threat-control beliefs, further amplify the risk of experiencing violence [17]. These delusions are characterized by the beliefs that certain people want to cause harm to the patient (threat) or that external forces control his/her mind (control/override) [18]. Research underscores the pervasive nature of non-war-related intimate and non-intimate partner interpersonal violence among individuals with severe mental illness; up to 56% of adults with severe mental illness have experienced some form of interpersonal violence [18]. With physical and sexual violence specifically among them being as high as 22% in females, 10% among males [13]. Studies across different regions, including Africa and high-income countries, consistently highlight high prevalence rates of violence against this vulnerable population [13,14,19]. For instance, in England, 16% of individuals with severe mental illness reported experiencing interpersonal violence [20]. Similarly, studies in Africa, such as those conducted in the Democratic Republic of Congo and Ethiopia, reveal alarmingly high prevalence rates (17.4% and 60.7%, respectively) of interpersonal violence among psychiatric patients [21,22].

People with severe mental illness are more vulnerable to physical and sexual violence due to certain other factors that increase their susceptibility [23,24]. Some of the factors contributing to the vulnerability include; unemployment, younger age, substance use, severity of psychiatric symptoms, non-adherence to treatment, history of violence perpetration, personality disorders, and homelessness [20–22,25–28]. Additional factors are impaired reality testing, disorganized thought processes, impulsivity, challenges with planning and problem-solving, socially disorganized living conditions, and substance use [11,29,30]. Gender disparities are evident, with younger males under 25 years at a higher risk of perpetrating violence, while females are more susceptible to being victims of physical and sexual violence [25]. Substance use significantly amplifies the risk of victimization among individuals with severe mental illness (SMI), and this has also been supported by studies linking substance use disorders to increased incidents of violence in this population [25,31]. Individuals who experiencing housing instability are homeless or face higher levels of violence compared to the general population [32]. There are significantly higher rates of physical assault and sexual victimization among homeless individuals compared to those with stable housing [29].

Studies have shown that mentally ill individuals with a history of aggression are at higher risk of experiencing violence: This vulnerability is often linked to their mental health conditions, which can impair their ability to manage conflicts and effectively communicate negative emotions and consequently, they may find themselves involved in situations that lead to violent victimization [18].

Interpersonal violence, including physical and sexual forms among individuals with severe mental illness has profound implications, including increased rates of relapse, post-traumatic stress disorder (PTSD), poor treatment outcomes, difficulty adhering to treatment, diminished quality of life, and poor functional and symptomatic recovery for the patient [14,18,22,32,33]. Traumatic victimization experiences correlate significantly with symptom severity and illness trajectory among psychiatric patients [34].

In Uganda, where about 35% of the population experiences some form of mental health challenge, individuals with severe mental illness often face human rights violations, including interpersonal violence [35,36]. Despite the profound impact of interpersonal violence on health and well-being, there is a significant gap in understanding its prevalence and associated risk factors among psychiatric outpatients in Uganda: Existing research in the country has predominantly concentrated on the mental health outcomes of violence, intimate partner violence, and violence affecting other vulnerable populations, including children with disabilities [37–39]. We still required information on reported interpersonal violence against people with severe mental illness. To that end, we hypothesized that; 1. compared to findings from existing research on the general population without severe mental illness (SMI), there is a higher prevalence of reported interpersonal violence among adult psychiatric out- patients receiving mental health care at Butabika National Referral Hospital; 2. known risk factors for reported interpersonal violence, such as co-morbid substance use, younger age, unstable housing, and male sex, would be associated with overall, physical, and sexual interpersonal violence among adult psychiatric out-patients receiving mental health care at Butabika National Referral Hospital; 3. Patients with a history of perpetrating physical or interpersonal violence are more likely to report being victims of physical, sexual, and overall interpersonal violence.

By filling this knowledge gap, our study aims to inform targeted interventions that can effectively mitigate the prevalence and impact of interpersonal violence among psychiatric patients, thereby promoting their safety and overall well-being. Achieving these objectives aligns with Sustainable Development Goals (SDGs) targets aimed at reducing violence-related mortality and promoting mental health globally [40,41].

## Materials and methods

### Study design, setting, and participants

From the 9^th^ of February 2020, we initiated a cross-sectional study targeting adults with severe mental illness receiving mental health care at the Butabika National Referral Hospital outpatient unit. This hospital is Uganda’s only mental health referral hospital and it is located in Butabika, a town that is approximately 12 kilometers east of Kampala’s central business district [42]. It offers daily outpatient clinic services for primary mental health care; it is a public facility [42]. As of 2019, Butabika National Referral Hospital catered to 25,360 mentally ill patients [42].

Recruitment of 385 study participants commenced on the 10^th^ of February 2020 and concluded on the 9^th^ of March 2020. The study involved participants who had received care for at least a year. This ensured that they had ample time to interact with mental health care professionals, receive a proper diagnosis, achieve some level of mental health stability (remission), and have proper documentation. The inclusion criteria were: 1. non-war related reported interpersonal violence perpetrated by a family member, intimate partner, friend, acquaintance, and/or stranger; 2. persons aged 18 years to 45 years and above whose Butabika National Referral Hospital records showed that they had been previously diagnosed with a severe mental illness (schizophrenia and other psychotic disorders, major depressive disorder and bipolar affective disorder); 3. Persons whom the clinicians at Butabika National Referral Hospital consider to be currently in remission as per the Diagnostic and Statistical Manual of Mental Disorders [43].

The study excluded patients who could not engage with the study process for whatever reason (experiencing active symptoms of their severe mental illness at the time of the study, too physically ill, having sensory impairment, and/ or having severe cognitive impairment); and patients who did not understand English or Luganda languages.

### Ethics approval and consent to participate

All methods were carried out in accordance with relevant institutional guidelines and regulations. The research protocol was approved by the Makerere University School of Medicine Research Ethics Committee under approval number #REC REF 2020–046. and we received administrative clearance to conduct the study was obtained from Butabika National Referral Hospital Research Ethics Committee. Written informed consent was obtained from each participant. Utmost confidentiality of all data collected was ensured using identification numbers and storing all data under lock and key including passwords for computer stored data.

### Measures

Participants completed a socio-demographic questionnaire that assessed their age, sex, religion, tribe, education level, employment status, and living situation. Mental illness diagnosis, co-morbid substance use, and the number of hospitalizations were obtained from the patient’s hospital documents. Severe Mental Illness (SMI) was defined as a condition in which an individual over the age of 18 had, within the past year, one or more of the following diagnosable mental disorders: schizophrenia and other psychotic disorders, major depressive disorder, or bipolar affective disorder. These disorders must cause serious functional impairment or require at least one hospital admission.

We adapted the WHO definition of violence for our study; It is important to note that while the WHO definition of violence includes not only physical force and sexual violence but also threatened force, psychological harm, and deprivation [2]. Our study primarily focuses on the physical and sexual aspects of interpersonal violence. This focus aligns with our objective of understanding the specific physical and sexual violence experienced by individuals with severe mental illness, which necessitate distinct health interventions. The operational definition used in this study is based on the modified My Exposure to Community Violence Questionnaire [44]. This encompasses actual physical and sexual violence. Interpersonal violence is defined as any physical or sexual harm inflicted upon a study participant by another person or persons: this encompasses actual acts of physical force that may lead to bodily injury, pain, or impairment, as well as non-consensual sexual acts or behaviors imposed through force, intimidation, or coercion.

We used the term "reported interpersonal violence" to refer to a self-reported experience of interpersonal violence by the study participant. Reported interpersonal violence was assessed using the modified My Exposure to Community Violence Questionnaire; which assesses lifetime and past year exposure to community violence [45]. We modified the existing scale by extracting the questions that pertained to actual physical and sexual violence. Left out questions pertaining to threatened violence. We also replaced the term "gun" with "clubs or stones," as these were more likely to be used as weapons in the Ugandan context. The tool had not been previously used in the local Ugandan setting. Therefore, it was translated and then back-translated by two independent teams of mental health workers fluent in both English and Luganda. The translated tool was then pre-tested among potential study participants, and it was observed that it was easy to comprehend and it took approximately 20 minutes to administer. The internal consistency of the modified tool was satisfactory at a Cronbach’s alpha of 0.63.

The tool comprised 9 questions evaluating the experience of violence in the 12 months leading up to the interview. Each question was divided into three parts: Part A assessed the history of experiencing physical or sexual violence; Part B evaluated the frequency of these violent acts, with categories including once, 2–3 times, 4–10 times, and more than 10 times; Part C identified the perpetrators of the violent acts, categorized as Peer, Partner, Family member, and Stranger. Overall reported interpersonal violence was scored as any positive response to questions 2A, 3A, 4A, 5A, 6A, and 7A. Physical violence was recorded as a positive response to any one of the questions 2A, 3A, 4A, and 5A, while sexual violence was a positive response to 6A and/ or 7A. From past literature, this tool has demonstrated good estimates of internal consistency for past year exposure to community violence [44]. We assessed the history of violence perpetration by the study participant over the past 12 months using a single question modified from a previous study. [46]; it read, “Have you ever hit, slapped, punched, shoved, choked, kicked, shaken, or otherwise physically hurt anyone else in the past 12 months.”

The sample size was estimated by using Leslie Kish (1965) formula for descriptive studies; using an estimate of 50% as proportion (P) of mentally ill patients that are victims of violent acts [47]. An apriori sample size estimation formula for multiple regression analysis was used for associated factors [48].

### Sampling procedure

The nursing officer in charge of the mental health unit introduced the principal investigator (PI) and his team of two research assistants (RAs), both psychiatric clinical officers, to the Butabika National Referral Hospital outpatient department staff and participants. The PI provided a short overview of the research. The RAs were well-trained on the study’s objectives, questionnaires, recruitment, and procedures and collected the data. The team reviewed the outpatient register daily using a systematic sampling approach, selecting patients in the order of their arrival at the clinic, with the earliest arrivals being recruited first. The mental health outpatient unit at Butabika National Referral Hospital serves an average of 2,113 patients per month, which equates to around 89 patients per day. The sample size was estimated by using Leslie Kish’s (1965) formula for descriptive studies, using an estimate of 50% as the proportion (P) due to lack of evidence on the mentally ill patients that are victims of violent acts [47]. Given the sample size of 385 participants (approximately 14 participants per day), the sampling interval was determined by dividing 89 by 14, resulting in 6.4, which we rounded off to 7. This sampling interval (n) was used to determine the next participant from the registry. If the 7^th^ patient in the sequence was not willing or unable to participate, then the next patient was approached. The research team was allocated 3 positions; 1^st^, 2^nd^, and 3^rd^. The participants were then allocated to the RAs in sequence, i.e., the first patient to the 1^st^ RA and the second patient to the 2^nd^ and so on, with the sequence repeating every after the third participant in line. Patients who completed their clinical review at the outpatient clinic were informed about a survey if their review coincided with the sampling interval from the hospital outpatient register and if they met the study’s eligibility criteria. The patients were assured that participating in the study or not would not affect their quality of treatment. After agreeing to participate, they were given a written informed consent form to read or have read to them by research assistants. Participants signed the form or inserted their thumbprint before the questionnaires were administered. Patients who needed help reading or understanding the consent form were offered assistance. This process was Witnessed by a relative of the participants. The consent forms and questionnaires were available in both English and Luganda. Study data was obtained through a review of the patient’s clinical file and the administration of the study questionnaire.

### Data management and analysis

Data was entered using Epi-data version 3.1, and statistical analysis was performed with STATA version 14. The characteristics of the study participants were computed to obtain frequencies, mean, standard deviation and percentages.

For the first objective, the proportion of participants experiencing reported interpersonal violence was computed and presented with a 95% confidence interval.

For the second objective, Simple logistic regression was used to determine the effect of the association between single exposure and the outcome of interest and was expressed as Odds Ratios. Independent variables with a p-value of <0.2 at bi-variable analysis were entered into the multi-variable model to determine independent factors associated with reported overall interpersonal violence; reported physical interpersonal violence, and reported sexual interpersonal violence. We assessed statistical differences between various demographic factors and reported interpersonal violence using bi-variable logistic regression. This initial analysis aimed to identify potential associations between individual demographic variables and the outcome variable. The results of the preliminary analysis guided the selection of relevant covariates for inclusion in the subsequent multi-variable logistic regression model. From the multi-variable model, factors whose p-values were <0.05 were considered statistically significant.

We used the Likelihood Ratio Test (LR chi-square) to assess the overall significance of the model. The mean variance inflation factor (VIF) was 1.19<10 which indicates that the predictor variables in the model did not exhibit any multi-collinearity.

During data collection, the missing variables were verified from the responsible research assistant, and where necessary the RA was sent back to the field to collect the missing information. At analysis, we adopted a multifaceted approach. For categorical variables, missing data were treated as a distinct category to preserve all available information. For continuous variables, we employed multiple imputation techniques to estimate missing values based on observed data patterns. This ensured that the inherent variability and relationships within the data-set were appropriately captured.

## Results

There were 385 study participants with a median age of 35 years (IQR: 28, 44), and about one-third (34%) of these respondents were aged between 25 to 34 years old. More than half of the study participants, 223 (approximately 60%) were female and 156 (approximately 41%) were never married, and 165 (43%) had secondary education. Almost two-thirds of the study participants were unemployed. About 57% which accounted for 216 participants, were living with friends and family, and more than two thirds were living in a household with 5 people or less.

Nearly half of the study participants had been diagnosed with bipolar disorder. The majority of the participants, about 68%, had their last active episode of mental illness more than 12 months prior. Close to a third, approximately 29%, had been admitted once in the past 12 months. About a tenth (13%) of the study participants reported co-morbid substance use and alcohol use disorder. See Table 1. Most of the respondents, 331 (86%), had no history of perpetrating physical violence in the past 1 year (Table 1).

**Table 1:** Association of background characteristics, clinical characteristics, and violence perpetration variables with overall interpersonal violence.

Variable	Prevalence of overall interpersonal violence			p-value
	Frequency (%)	Yes n (%)	No n (%)	
**Age**				
18–24	56 (14.6)	30 (22.7)	26 (10.3)	**0.001**
25–34	131 (34)	51 (38.6)	80 (31.6)	
35–44	103 (26.7)	29 (22.0)	74 (29.2)	
45+	95 (24.7)	22 (16.7)	73 (28.9)	
**Sex**				
Male	162 (42.1)	52 (39.4)	110 (43.5)	0.441
Female	223 (57.9)	80 (60.6)	143 (56.5)	
**Religion**				
Catholic	125 (32.5)	46 (34.8)	79 (31.2)	**0.132**
Protestant	197 (51.2)	59 (44.7)	138 (54.5)	
Muslims	63 (16.3)	27 (20.5)	36 (14.2)	
Marital Status (n = 381) *				
Married/Cohabiting	120 (31.5)	31 (23.7)	89 (35.6)	**0.085**
Never married	156 (40.9)	58 (44.3)	98 (39.2)	
Widowed	19 (5)	6 (4.6)	13 (5.2)	
Separated/Divorced	86 (22.6)	36 (27.5)	50 (20.0)	
Education level (n = 383) *				
No formal education	11 (2.9)	4 (3.1)	7 (2.8)	0.329
Primary education	133 (34.7)	51 (38.9)	82 (32.5)	
Secondary education	165 (43.1)	57 (43.5)	108 (42.9)	
Tertiary education	74 (19.3)	19 (14.5)	55 (21.8)	
Employment Status (n = 384) *				
Unemployed	230 (59.9)	89 (67.4)	141 (56.0)	**0.029**
Employed	154 (40.1)	43 (32.6)	111 (44.0)	
Housing situation (n = 381) *				
No permanent residence	25 (6.6)	15 (11.4)	10 (4.0)	**0.001**
Owning/Renting a home	140 (36.7)	34 (25.8)	106 (42.6)	
Living with friends and family	216 (56.7)	83 (62.9)	133 (53.4)	
**Number of people in a household**				
≤5	268 (69.6)	97 (73.5)	171 (67.6)	0.233
>5	117 (30.4)	35 (26.5)	82 (32.4)	
**Co-morbid substance use**				
No	335 (87)	113 (85.6)	222 (87.7)	0.553
Yes	50 (13)	19 (14.4%)	31 (12.3)	
**Type of substance use disorder**				
Alcohol use disorder	50 (13)	18 (13.6)	32 (12.6)	0.232
Cannabis use disorder	6 (1.6)	0 (0.0)	6 (2.4)	
None	329 (85.5)	114 (86.4)	215 (85.0)	
**Mental illness diagnosis**				
Depressive disorder	48 (12.5)	13 (9.8)	35 (13.8)	0.499
Schizophrenia	118 (30.7)	46 (34.8)	72 (28.5)	
Bipolar	191 (49.6)	64 (48.5)	127 (50.2)	
Other psychotic disorders	28 (7.3)	9 (6.8)	19 (7.5)	
**Last active episode of mental illness (n = 185)**				
≤12 months	60 (32.4)	23 (31.5)	37 (33.0)	0.828
>12 months	125 (67.6)	50 (68.5)	75 (67.0)	
**Admissions past 12 months**				
No admissions	236 (61.3)	79 (59.8)	157 (62.1)	0.868
Once	113 (29.4)	41 (31.1)	72 (28.5)	
Twice and more	36 (9.4)	12 (9.1)	24 (9.5)	
**Participant perpetrated physical violence**				
No	331 (86)	89 (67.4)	242 (95.7)	**<0.001**
Yes	54 (14)	43 (32.6)	11 (4.3)	
**Frequency of Perpetrating physical Violence (n = 54)**				
Once	28 (51.8)	22 (50.0)	6 (60.0)	0.555
2–3 times	14 (25.9)	13 (29.5)	1 (10.0)	
4–10 times	7 (13)	5 (11.4)	2 (20.0)	
>10 times	5 (9.3)	4 (9.1)	1 (10.0)	
**Victims of physical violence**				
Peer	6 (13.3)	6 (16.7)	0 (0.0)	0.536
Partner	4 (8.9)	3 (8.3)	1 (11.1)	
Family member	25 (55.6)	20 (55.6)	5 (55.6)	
Stranger	10 (22.2)	7 (19.4)	3 (33.3)	

*Missing data due to participants declining to respond to questions.

The emboldened p-values are those that reach the level of statistical significance.

About a third of the respondents, 132 (about 34%), reported experiencing some form of interpersonal violence. Physical interpersonal violence was reported by 113 (approximately 29%), with about 2/3 reported experiencing one episode in the last one year. Sexual interpersonal violence was reported by 43 participants (a little over 11%), with 60% of them experiencing a single episode in the last one year (Table 2). Most of the respondents, 331(86%), had no history of perpetrating physical violence in the past 1 year (Table 2).

**Table 2:** Report of experiencing and perpetrating interpersonal violence by the study.

Variable	Frequency (n = 385)	Percentage (%)
**Overall reported interpersonal violence**		
No	253	65.7
Yes	132	34.3
**Experienced Sexual violence**		
No	342	88.8
Yes	43	11.2
**Experienced Physical violence**		
No	272	70.6
Yes	113	29.4
**Frequency of physical violence**	**n = 113**	
Once	72	63.7
2–3 times	30	26.6
4–10 times	10	8.8
>10 times	1	0.9
**Frequency of sexual violence**	**n = 40**	
Once	24	60.0
2–3 times	16	40.0
**Perpetrated physical violence**		
No	331	86.0
Yes	54	14.0
**Frequency of Perpetrating physical Violence**		
Once	28	51.8
2–3 times	14	25.9
4–10 times	7	13.0
>10 times	5	9.3
**Victims of physical violence**		
Peer	6	13.3
Partner	4	8.9
Family member	25	55.6
Stranger	10	22.2

In bivariable analyses, as shown in Table 1, background characteristics that were associated with reported interpersonal violence at a threshold of p<0.2 were: age, religion, marital status, employment status, and living situation.

The clinical characteristics and violence perpetration variable that were associated with reported interpersonal violence at bivariable analyses, were; type of substance use disorder and a history of perpetrating physical violence (Table 1).

All the factors that attained a threshold of p<0.2 at bivariable analyses were entered into the multivariate model. The factors that were significantly independently associated with overall reported interpersonal violence were: 1. older age which was protective, compared to those aged 18–24 years; those aged 35–44 years had adjusted odds ratio [49] = 0.31 (95% confidence interval (CI): 0.14–0.70, p = 0.005) while those aged 45 years and above had AOR = 0.34 (95% CI: 0.15–0.80, p = 0.013). 2. Having perpetrated physical violence significantly increased the odds of experiencing overall reported interpersonal violence AOR = 9.64 (95% CI: 4.49–20.67, p = <0.001) (Table 3).

**Table 3:** Factors associated with reported overall interpersonal violence at multivariable analyses.

Variable	Overall interpersonal violence		Crude odds ratios (95% (CI)	P value	Adjusted odds ratios (95% CI)	p value
	n (%)					
	Yes	No				
**Age**						
18–24	30 (22.7)	26 (10.3)	1		1	
25–34	51 (38.6)	80 (31.6)	0.55 (0.29–1.04)	0.066	0.62 (0.28–1.12)	0.189
35–44	29 (22.0)	74 (29.2)	0.34 (0.17–0.66)	0.002	0.31 (0.14–0.70)	**0.005**
45+	22 (16.7)	73 (28.9)	0.26 (0.13–0.53)	<0.001	0.34 (0.15–0.80)	**0.013**
**Sex**						
Male	52 (39.4)	110 (43.5)	1			
Female	80 (60.6)	143 (56.5)	1.18 (0.77–1.82)	0.441		
**Religion**						
Catholics	46 (34.8)	79 (31.2)	1		1	
Protestants	59 (44.7)	138 (54.5)	0.73 (0.46–1.18)	0.202	0.76 (0.44–1.31)	0.369
Muslims	27 (20.5)	36 (14.2)	1.29 (0.69–2.4)	0.422	1.53 (0.75–3.12)	0.319
**Marital status**						
Married/cohabiting	31 (23.7)	89 (35.6)	1		1	
Never married	58 (44.3)	98 (39.2)	1.70 (1.01–2.86)	0.047	0.87 (0.45–1.70)	0.691
Widowed	6 (4.6)	13 (5.2)	1.33 (0.46–3.79)	0.599	0.96 (0.29–3.21)	0.95
Separated/ Divorced	36 (27.5)	50 (20.0)	2.07 (1.14–3.74)	0.016	1.44 (0.70–2.92)	0.32
**Employment status**						
Unemployed	89 (67.4)	141 (56.0)	1		1	
Employed	43 (32.6)	111 (44.0)	0.61 (0.39–0.95)	0.03	0.79 (0.46–1.34)	0.375
**Housing situation**						
No permanent residence	15 (11.4)	10 (4.0)	1		1	
Owning/Renting a home	34 (25.8)	106 (42.6)	0.21 (0.09–0.52)	0.001	0.50 (0.17–1.48)	0.208
Living with friends and family	83 (62.9)	133 (53.4)	0.42 (0.18–0.97)	0.042	0.79 (0.29–2.15)	0.647
**Number of people in a household**						
≤5	97 (73.5)	171 (67.6)	1		1	
>5	35 (26.5)	82 (32.4)	0.75 (0.47–1.20)	0.203	0.87 (0.50–1.50)	0.618
**Last episode of active mental illness**						
≤12 months	23 (31.5)	37 (33.0)	1			
s>12 months	50 (68.5)	75 (67.0)	1.07 (0.57–2.02)	0.828		
**Type of substance use disorder**						
Alcohol use disorder	18 (13.6)	32 (12.6)	1			
Cannabis use disorder	0 (0.0)	6 (2.4)	-	-		
None	114(86.4)	215 (85.0)	0.94 (0.51–1.75)	0.852		
**Admitted in the past 12 months**						
No admissions	79 (59.8)	157 (62.1)	1			
Once	41 (31.1)	72 (28.5)	1.13 (0.71–1.81)	0.605		
Twice and more	12 (9.1)	24 (9.5)	0.99 (0.47–2.09)	0.987		
**Perpetrated physical violence**						
No	89 (67.4)	242 (95.7)	1		1	
Yes	43 (32.6)	11 (4.3)	10.63(5.25–21.5)	<0.001	9.64 (4.49–20.67)	**<0.001**

The emboldened p-values are those that reach the level of statistical significance.

Variables with p-values > 0.2 in the crude analysis were not further adjusted, and therefore, adjusted odds ratios (AORs) are not presented for those variables.

In a sub-analysis looking at the factors associated with physical violence and sexual violence separately; about reported physical interpersonal violence, older age was protective; compared to those aged 18–24 years, participants aged 35–44 years had AOR = 0.33 (95% CI: 0.14–0.76, p = 0.010) while those aged 45+ years had AOR = 0.24 (95% CI: 0.09–0.61, p = 0.003). The odds of physical reported interpersonal violence were 13.7 times higher among individuals that had a history of perpetrating physical violence compared to those that had not (AOR = 13.68, 95% CI: 6.31–29.64, p = <0.05) (Table 4).

**Table 4:** Factors associated with reported physical violence at multivariable analyses.

Variable	Prevalence of Physical violence		Crude odds ratios (95% CI)	p value	Adjusted odds ratios (95% CI)	P value
	Yes. N (%)	No. n (%)				
**Age**						
18–24	26 (23.0)	30 (11.3)	1		1	
25–34	44 (38.9)	87 (32.7)	0.58 (0.31–1.10)	0.098	0.63 (0.29–1.33)	0.225
35–44	27 (23.9)	76 (28.6)	0.41 (0.21–0.81)	0.011	0.33 (0.14–0.76)	**0.01**
45+	16 (14.2)	73 (27.4)	0.23 (0.11–0.50)	<0.001	0.24 (0.09–0.61)	**0.003**
**Sex**						
Male	44 (38.9)	118 (43.7)	1			
Female	69 (61.1)	152 (56.3)	1.20 (0.77–1.88)	0.421		
**Religion**						
Catholics	42 (37.2)	83 (30.5)	1		1	
Protestants	48 (42.5)	149 (54.8)	0.63 (0.39–1.04)	0.073	0.62 (0.35–1.11)	0.369
Muslims	23 (20.4)	40 (14.7)	1.14 (0.60–2.14)	0.692	1.28 (0.61–2.69)	0.319
**Marital status**						
Married/ cohabiting	25 (22.3)	95 (35.3)	1		1	
Never married	48 (42.9)	108 (40.1)	1.69 (0.97–2.95)	0.065	0.87 (0.42–1.80)	0.713
Widowed	6 (5.4)	13 (4.8)	1.75 (0.61–5.08)	0.3	1.49 (0.41–5.32)	0.543
Separated /Divorced	33 (29.5)	53 (19.7)	2.37 (1.27–4.39)	0.006	1.78 (0.84–3.78)	0.136
**Employment status**						
Unemployed	79 (69.9)	151 (55.7)	1		1	
Employed	34 (30.1)	120 (44.3)	0.54 (0.34–0.86)	0.01	0.66 (0.37–1.17)	0.155
**Housing situation**						
No permanent residence	12 (10.6)	13 (4.9)	1		1	
Owning/ Renting a home	29 (25.7)	111 (41.4)	0.28 (0.12–0.68)	0.005	0.94 (0.30–3.00)	0.914
Living with friends and family	72 (63.7)	144 (53.7)	0.54 (0.18–0.97)	0.15	1.32 (0.46–3.77)	0.604
**Number of people in a household**						
≤5	84 (74.3)	184 (67.6)	1		1	
>5	29 (25.7)	88 (32.4)	0.72 (0.44–1.18)	0.195	0.87 (0.49–1.56)	0.646
**Type of substance use disorder**						
Alcohol use disorder	16 (14.2)	34 (12.5)	1			
Cannabis use disorder	0 (0.0)	6 (2.2)	-	-		
None	97 (85.8)	232 (85.3)	0.89 (0.47–1.68)	0.717		
**Last episode of active mental illness**						
≤12 months	17 (28.3)	43 (34.4)	1			
>12 months	43 (71.7)	82 (65.6)	1.33 (0.68–2.60)	0.410		
**Admitted in the past 12 months**						
No admissions	72 (63.7)	164 (60.3)	1			
Once	31 (27.4)	82 (30.1)	0.86 (0.52–1.41)	0.556		
Twice and more	10 (8.8)	26 (9.6)	0.88 (0.40–1.91)	0.740		
**Perpetrated physical violence**						
No	70 (61.9)	261 (96.0)	1		1	
Yes	43 (38.1)	11 (4.0)	14.60 (7.15–29.73)	<0.001	13.68(6.31–29.64)	**<0.001**

The emboldened p-values are those that reach the level of statistical significance.

Variables with p-values > 0.2 in the crude analysis were not further adjusted, and therefore, adjusted odds ratios (AORs) are not presented for those variables.

Looking at reported sexual interpersonal violence, all the factors that attained a threshold of p<0.2 at bi-variable analyses were entered into the multivariate model. We found that compared to those who had no permanent residence, owning or renting a home decreased the odds of experiencing sexual reported interpersonal violence AOR = 0.17 (95% CI: 0.03–1.04 p = 0.05). Admission to hospital in the past 12 months increased the odds of experiencing sexual reported interpersonal violence AOR = 3.04 (95% CI: 0.99–9.31, p = 0.05) (Table 5).

**Table 5:** Factors associated with reported sexual violence at multivariable analyses.

Variable	Sexual violence		Crude odds ratios (95% CI)	p value	Adjusted odds ratios (95% CI)	p value
	Yes	No				
**Age**						
18–24	8 (18.6)	48 (14.0)	1			
25–34	17 (39.5)	114 (33.3)	0.89 (0.36–2.21)	0.810		
35–44	10 (23.3)	93 (27.2)	0.65 (0.24–1.74)	0.387		
45+	8 (18.6)	87 (25.4)	0.55 (0.19–1.56)	0.263		
**Sex**						
Male	15 (34.9)	147 (43.0)	1			
Female	28 (65.1)	195 (57.0)	1.41 (0.73–2.73)	0.312		
**Religion**						
Catholics	12 (27.9)	113 (33.0)	1			
Protestants	22 (51.2)	175 (51.2)	1.18 (0.56–2.49)	0.656		
Muslims	9 (20.9)	54 (15.8)	1.57 (0.62–3.95)	0.339		
**Marital status**						
Married/cohabiting	12 (27.9)	108 (32.0)	1			
Never married	19 (44.2)	137 (40.5)	1.24 (0.58–2.68)	0.570		
Widowed	2 (4.7)	17 (5.0)	1.06 (0.22–5.15)	0.944		
Separated/Divorced	10 (23.3)	76 (22.5)	1.18 (0.49–2.88)	0.709		
**Employment status**						
Unemployed	28 (65.1)	202 (59.2)	1			
Employed	15 (34.9)	139 (40.8)	0.78 (0.40–1.51)	0.459		
**Housing situation**						
No permanent residence	19 (33.9)	6 (1.8)	1		1	
Owning/Renting a home	8 (14.3)	132 (40.6)	0.19 (0.06–0.61)	0.005	0.17 (0.03–1.04)	**0.05**
Living with friends and family	29 (51.8)	187(57.5)	0.49 (0.18–1.33)	0.162	0.91 (0.26–3.13)	0.88
**Number of people in a household**						
≤5	31 (72.1)	237 (69.3)	1			
>5	12 (27.9)	105 (30.7)	0.87 (0.43–1.77)	0.707		
**Type of substance use disorder**						
Alcohol use disorder	5 (11.6)	45 (13.1)	1			
Cannabis use disorder	0 (0.0)	6 (1.8)	-	-		
None	38 (88.4)	291 (85.1)	1.18 (0.44–3.14)	0.748		
**Last active episode of mental illness**						
≤12 months	12 (48.0)	48 (30.0)	1		1	
>12 months	13 (52.0)	112 (70.0)	0.46 (0.20–1.09)	0.078	0.88 (0.30–2.59)	0.809
**Admitted in the past 12 months**						
No admissions	21 (48.8)	215 (62.9)	1		1	
Once	19 (44.2)	94 (27.5)	2.07 (1.06–4.03)	0.032	3.04 (0.99–9.31)	**0.051**
Twice and more	3 (7.0)	33 (9.6)	0.93 (0.26–3.29)	0.911	1.45 (0.15–14.51)	0.752
**Perpetrated physical violence**						
No	31 (72.1)	300 (87.7)	1		1	
Yes	12 (27.9)	42 (12.3)	2.76 (1.32–5.80)	0.007	1.61 (0.55–4.68)	0.381

The emboldened p-values are those that reach the level of statistical significance.

Variables with p-values > 0.2 in the crude analysis were not further adjusted, and therefore, adjusted odds ratios (AORs) are not presented for those variables.

## Discussion

In this study, we proposed three hypotheses regarding the prevalence and risk factors for interpersonal violence among adult psychiatric out-patients receiving mental health care at Butabika National Referral Hospital over a period of 12 months. First, we hypothesized that the prevalence of reported interpersonal violence would be higher in this population compared to findings from existing research on the general population without severe mental illness (SMI). Second, we posited that known risk factors, such as co-morbid substance use, younger age, unstable housing, and male sex, would be significantly associated with overall, physical, and sexual interpersonal violence. Finally, we anticipated that patients with a history of perpetrating physical or interpersonal violence would be more likely to report being victims of physical, sexual, and overall interpersonal violence. The following discussion evaluates these hypotheses considering the study’s results, comparing our findings with existing literature and considering the implications for clinical practice and future research.

We found that the prevalence of overall interpersonal violence among individuals with severe mental illness was 34.3%. Specifically, the prevalence of physical interpersonal violence was approximately 29%, and sexual interpersonal violence was reported at just above 11%. These figures are notably higher than those from a previous research looking at violence among the general Ugandan population, as we had originally hypothesized [50]. The findings above showed that individuals with severe mental illness in the low-income country of Uganda experience high levels of interpersonal violence. However, this issue is not unique to Uganda and is also prevalent in high-income countries [19]. Previous research showed that there is a higher likelihood of individuals with severe mental illness experiencing violence compared to the general population [14,15]. Violence among this population may be amplified by psychotic symptoms, such as delusions involving threat-control beliefs: These delusions are characterized by the belief that certain individuals wish to cause harm to the patient (threat) or that external forces are controlling or overriding their thoughts (control/override) [17,18].

The prevalence of overall interpersonal violence among people with severe mental illness in our study (34.3%) was comparable to the 35% reported in a previous study conducted in the USA among individuals with SMI [19]. The prevalence of reported physical interpersonal violence in our study (29%) was similar to the 29% reported in a study conducted among patients with SMI across multiple European countries, including the Czech Republic, Germany, Lithuania, Poland, Slovakia, and Sweden [28]. In connection to this, a systematic review regarding the occurrence of physical or sexual abuse among individuals with disabilities suggested that approximately 25% of those with severe mental illness have encountered physical violence in recent times [11].

Based on our research, the reported incidence of sexual interpersonal violence is just above 11%, which is slightly lower than the 15% reported in a previous cross-sectional study conducted among the Swedish population [51].

This is perhaps because sexual assault in the sub-Saharan African psychiatric settings is often under-reported due to challenges faced by both patients and staff [52,53]. One reason for under-reporting of rape and sexual assault could be the stigma and societal taboos surrounding these events, which may discourage victims from seeking medical attention or reporting the crime [54–56]. Patients and staff may be hesitant to report incidents due to various reasons such as stigma, guilt, mistrust, powerlessness, fear of retaliation, and disbelief; Staff may also be hesitant due to concerns about legal implications, reliability of allegations, a culture of silence, or disbelief of any such incident happening [53,57].

Our findings revealed that a number of the factors we hypothesized, such as younger age, a history of perpetrating physical interpersonal violence, unstable housing, and recent hospital admissions, were indeed associated with various forms of violence among adult psychiatric out-patients at Butabika National Referral Hospital. We found that the odds of experiencing overall interpersonal violence decrease as one gets older. Previous studies have reported similar findings [18,20,26–28,55]. This may be due to older individuals having more stable and selective social interactions that help avoid or quickly resolve conflicts. Additionally, their tendency to stay at home more often than younger people may further reduce their exposure to violent victimization [58–61]. Also, it is commonly believed that older people are more vulnerable and less able to protect themselves; this perception may make them less likely to be victims of violence [62].

This study also discovered that participants who had a record of perpetrating physical interpersonal violence were more likely to report being victims of physical, overall interpersonal violence. Their likelihood of reporting physical interpersonal violence also increased significantly, up to 13.7 times higher among them. In recent systematic review conducted by Latalova et al, there is strong evidence indicating a connection between a history of perpetrating interpersonal violence and experiencing it [18]. Studies conducted in the UK and Ethiopia had similar observations [17,22]. This relationship may be due to the impact of severe mental illness on an individual’s ability to manage conflicts and communicate negative emotions effectively; such impairments can increase the likelihood of both engaging in and experiencing violence, making individuals more vulnerable to victimization [18]. Silver proposed another explanation for disturbed or psychotic behavior in acutely ill patients; he noted that this behavior can elicit hostile reactions and attempts at social control from others, potentially resulting in conflict and mutual violence [63].

After adjusting for other factors, individuals aged 45 years and above had significantly lesser odds of physical violence compared to individuals aged 18 to 24 years. Also, those aged between 35 to 44 years had significantly lesser odds of physical violence compared to those aged between 18 to 24 years. These findings revealed that the younger a participant is, the higher the likelihood of experiencing physical violence. This is similar to what other researchers found in the past [15]. Our findings also concur with those indicated by previous studies conducted among patients with mental disorders in South America which found that younger age was significantly associated with physical violence [28,64,65]. This may be because older individuals often spend more time at home compared to younger people, they might be less exposed to violence [58,59]. The odds of physical violence were observed to be significantly 20 times higher among individuals that have ever physically abused another person compared to those that have not. This association between experiencing violence and history of violence perpetration is consistent with another study [66]. This may also be owed to the fact that the difficulties that individuals with severe mental illness face in managing conflicts and communicating negative emotions effectively may contribute to their increased vulnerability to both perpetrating and experiencing violence: These challenges can make it harder for them to navigate potentially harmful situations, thereby raising their risk of victimization [18].

Regarding reported sexual interpersonal violence, we observed a prevalence of up to 11% of the study participants. Not owning a permanent home and having been admitted to the hospital in the past 12 months were associated with sexual violence. Individuals owning or renting a home had significantly 83% lesser odds of sexual violence compared to those that had no permanent residence. Similar findings were shown in past research [29]. Owning or renting a home offers individuals a stable and controlled living environment, which can significantly reduce their vulnerability to sexual violence: Stability in housing means individuals are less likely to be exposed to risky environments where violence is more likely to occur [32]. In contrast, those without a permanent residence, often face precarious living conditions; this includes overcrowded housing, lack of privacy, and dependence on others for shelter, which can increase their risk of experiencing sexual violence [32,67].

We also found that the odds of sexual violence were higher among individuals that had been admitted once in the past 12 months compared to those that had not been admitted. Patients may be vulnerable to sexual violence while admitted to mental health facilities; up to 45% of women experience a sexual assault during an inpatient admission; men and women with mental disorders who have a history of being hospitalized for psychiatric treatment in the past are more likely to experience sexual violence [65,68,69]. This may be because severe mental illnesses, such as those presenting with mania and psychosis, experienced by inpatients are a major contributor to sexual assaults in psychiatric facilities [69]. These conditions often cause a lack of awareness, impaired judgment, and hyper-sexuality, making patients vulnerable to exploitation [69]. Psychiatric hospitals, including Butabika, often have inadequate staffing and poor patient supervision plus outdated and poorly designed structures, which contribute to low visibility from the nurses’ station [69,70]. A study in the UK found that over 13% of psychiatric patients exhibited sexual behavior within the first 2 weeks of their stay, primarily involving indecent exposure and non-consensual touching [71].

The limitations of this study included: 1) Inability of the study to assess the direction of causality due to its cross-sectional nature, 2) There could have been recall bias for events that had happened previously 12 months. We mitigate recall bias by cross-referencing participant records with their self-reported data to verify and fill gaps, offering reviews for discrepancies without directly showing records to participants, and maintaining strict confidentiality and ethical standards throughout the process, 3) It is possible that the study suffered from selection bias, as only patients in remission were included, and all participants were outpatients; also, those who were unable to engage were excluded. This may have resulted in a lack of representation for certain conditions of individuals with mental illness. The involvement of relatives in witnessing the consenting process could have been a potential source of selection bias. Relatives who were aware of or involved in the violence might have influenced the participants’ willingness to disclose their experiences fully or to participate in the study due to fear of further violence or retribution. This could have affected the representativeness of the sample and the validity of the findings, and 4) We employed the My Exposure to Community Violence Questionnaire for the first time in this setting. While we were not able to undertake a full validation exercise on it, we were able to undertake forward and back translation to determine its reliability/internal consistency.

## Conclusion

This study revealed a significant prevalence of interpersonal violence among adults with severe mental illness receiving treatment at Butabika National Referral Hospital. Specifically, about a third of participants reported experiencing interpersonal violence. These rates are consistent with those observed in high-income countries [19,28], indicating that severe mental illness is associated with high levels of violence across different economic contexts.

Our findings also highlight that younger individuals with severe mental illness are more likely to experience interpersonal violence than older ones. This may be due to older individuals having more stable and selective social interactions that help avoid or quickly resolve conflicts: Additionally, their tendency to stay at home more often than younger people may further reduce their exposure to violent victimization [58–61]. Furthermore, the study identified that individuals with a history of violent behavior are significantly more likely to report being victims of violence themselves [17,22]. This suggests a troubling cycle of aggression and victimization, reinforcing patterns observed in other studies where past violent behavior correlates with an increased likelihood of experiencing violence [17].

Also, there was a pronounced vulnerability of younger individuals with severe mental illness to physical violence, echoing trends seen in previous research [14,27,64,65]. This may be because older individuals often spend more time at home compared to younger people, they might be less exposed to violence [58,59]. Additionally, the study also highlights a strong link between a history of violent behavior and the likelihood of experiencing physical violence, which is consistent with findings from research [66]. Participants with a history of perpetrating violence were 20 times more likely to report being victims of physical violence themselves. This also suggests a recurring pattern, where individuals who have engaged in violence are more likely to experience violence themselves, possibly due to persistent difficulties in managing conflicts and expressing negative emotions effectively [17].

Additionally, factors such as lack of permanent housing and recent psychiatric hospital admissions were associated with a higher likelihood of sexual violence. Individuals without stable housing and those who had been admitted to the hospital in the past 12 months were more vulnerable to sexual violence, likely due to overcrowded living conditions and increased susceptibility in psychiatric settings [69].

We propose some recommendations from our findings including: Implementing screening for interpersonal violence among young people visiting the Butabika National Referral Hospital outpatient department. This screening aims to identify any history of experiencing violence and its potential physical and psychological impacts on patients. Additionally, we suggest incorporating information on preventing the perpetration of physical violence and its consequences into the psycho-education provided to adults receiving care at Butabika National Referral Hospital.

A prospective study design is necessary to determine the causal relationship between reported interpersonal violence and various risk or protective factors. Qualitative studies are needed to gain a deeper understanding of reported interpersonal violence among individuals with severe mental illness. Furthermore, it is crucial to develop evidence-based interventions tailored to address interpersonal violence among individuals with mental illness.

The Ugandan Ministry of Health should consider addressing the perpetrators of violence against individuals with mental illness, possibly through mass sensitization campaigns highlighting the dangers and consequences of violence. These campaigns could have a multifaceted impact: directly reducing the risk of violence against SMI patients, fostering a more tolerant and supportive environment, and promoting empathy by educating the public about mental illness and dispelling misconceptions [72].

Community-based support programs should be established specifically targeting individuals at risk of interpersonal violence [73,74]. These programs could involve community outreach, peer support networks, and skill-building activities to enhance coping mechanisms and resilience.

## Supporting information

S1 Dataset (XLS)
